# Nivolumab and ipilimumab combination treatment in advanced dMMR/MSI-H non-colorectal cancers: Results from a phase II trial

**DOI:** 10.1001/jamaoncol.2025.4721

**Published:** 2026-01-01

**Authors:** Matteo S Carlino, Bo Gao, Michael Michael, Henry Marshall, Ashray Gunjur, Howard Chan, Rob Zielinski, Jane So, Samuel J Harris, Damien Kee, Ian M Collins, Wei-Sen Lam, Megan Lyle, Craig Underhill, Michael P Brown, Rosemary Harrup, Shu-Fen Wong, John Grady, Mandy Ballinger, Elnaz Tavancheh, David M Thomas, Jodie Palmer, Kylie Wilkie, Jonathan Cebon, Oliver Klein

**Affiliations:** Department of Medical Oncology, Blacktown and Westmead Hospitals, Sydney, Australia; Melanoma Institute of Australia, University of Sydney, Sydney, Australia; Department of Medical Oncology, Blacktown and Westmead Hospitals, Sydney, Australia; Department of Medical Oncology, Peter MacCallum Cancer Centre, Melbourne, Australia; Wellcome Sanger Institute, Hinxton, UK; Department of Medical Oncology, Calvary Mater Hospital, Newcastle, Australia; Central West Cancer Care Centre, Orange Base Hospital, Orange, Australia; Department of Medical Oncology, Auckland City Hospital, Auckland, New Zealand; Department of Medical Oncology, Bendigo Health, Bendigo, Australia; Department of Medical Oncology, Peter MacCallum Cancer Centre, Melbourne, Australia; Department of Medical Oncology, Austin Health, Melbourne, Australia; Southwest Healthcare, Warrnambool, Australia; School of Medicine, Deakin University, Geelong, Australia; Department of Medical Oncology, Fiona Stanley Hospital, Perth, Australia; Cancer Care Centre, Queensland Health, Cairns, Australia; Border Medical Oncology Research Unit, Albury-Wodonga Regional Cancer Centre, Albury-Wodonga, Australia; University of NSW School of Clinical Medicine, Rural Clinical Campus, Albury, New South Wales, Australia; John Richards Centre for Rural Ageing Research, Latrobe University, Wodonga, Australia; Department of Medical Oncology, Royal Adelaide Hospital, Adelaide, Australia; Cancer and Blood Services, Royal Hobart Hospital, Hobart, Australia; Department of Medical Oncology, University Hospital, Geelong, Australia; Centre for Molecular Oncology, University of New South Wales, Sydney, Australia; Olivia Newton-John Cancer Research Institute, Melbourne, Australia; Centre for Molecular Oncology, University of New South Wales, Sydney, Australia; Olivia Newton-John Cancer Research Institute, Melbourne, Australia; School of Translational Medicine, Monash University, Melbourne, Australia; Department of Medical Oncology, Austin Health, Melbourne, Australia; Olivia Newton-John Cancer Research Institute, Melbourne, Australia; School of Cancer Medicine, La Trobe University, Bundoora, Australia; School of Translational Medicine, Monash University, Melbourne, Australia

## Abstract

**Importance:**

Mismatch repair deficient (dMMR)/microsatellite instability-high (MSI-H) cancers constitute one of the most immunogenic malignancies. Anti-PD-1 monotherapy provides durable responses in a third of patients with advanced dMMR/MSI-H non-colorectal cancers (CRC). Combined anti-PD-1/CTLA-4 blockade using nivolumab and ipilimumab has shown superiority to anti-PD-1 monotherapy in other immunogenic cancers such as metastatic melanoma. MoST-CIRCUIT is the first trial that investigated combined anti-PD-1/CTLA-4 blockade in advanced dMMR/MSI-H non-colorectal cancers (CRC).

**Objective:**

To evaluate the efficacy and safety of combined anti-PD-1/CTLA-4 blockade using nivolumab and ipilimumab in advanced dMMR/MSI-H non-colorectal cancers.

**Design:**

The MoST-CIRCUIT prospective multicenter phase 2 non-randomized phase clinical trial enrolled patients with advanced dMMR/MSI-H into cohort D.

**Setting:**

Patients were enrolled from August 2021 to February 2024 across 17 Australian and New Zealand sites.

**Participants:**

52 Patients with advanced non-colorectal dMMR/MSI-H

**Interventions:**

Patients received nivolumab 3mg/kg and ipilimumab 1mg/kg q3 weekly for four doses, followed by nivolumab 480mg q4 weekly for 96 weeks, until disease progression or the development of unacceptable toxicity.

**Main outcome and Measures:**

Co-primary endpoints were objective response rate (ORR) and 6 month-progression free-survival (6-PFS) as assessed by RECIST1.1, with the secondary endpoints being median overall survival (mOS), progression-free survival (PFS) and treatment related toxicity.

**Results:**

52 patients representing 17 tumour types were enrolled with the most common tumour type being endometrial cancer (50%). 27 patients (52%) were pre-treated for metastatic disease. ORR was 63% (95% CI: 50 to 75%) with the median duration of response not being reached and 79% of responses being ongoing. The median PFS and OS have not been reached and the 6-month-PFS is 71% (95% CI: 57-81%). 23% of patients experienced a grade 3/4 immune-related adverse event.

**Conclusions and Relevance:**

Combined anti-PD-1/CTLA-4 blockade leads to a high rate of durable responses in dMMR/MSI-H non-CRC cancers comparing favourably to published trials using anti-PD-1/PD-L1 monotherapy. Anti-PD-1/CTLA-4 blockade using nivolumab and ipilimumab represents an alternative treatment option to monotherapy in this patient population.

**Trial Registration:**

Clinical Trials.gov registration: NCT04969887

## Introduction

Mismatch repair deficient (dMMR)/microsatellite unstable cancers (MSI-H) are highly immunogenic, demonstrating one of the highest response rates to anti-PD-1/PD-L1 checkpoint inhibitor therapy across tumour types.^1^ Microsatellite instability occurs on the background of genetic and epigenetic alterations affecting the expression and/or function of one of the four mismatch repair proteins (MLH1/PMS2/MSH2/MSH6).

This leads to genomic instability and is frequently accompanied by a high tumour mutational burden (TMB) contributing to the immunogenicity of these cancers.^2^ The frequency of microsatellite instability differs across tumour types and is most commonly seen in colorectal, endometrial and gastric cancers.^3^

In keeping with the high immunogenicity of dMMR/MSI-H cancers, checkpoint inhibition using anti-PD-1/PD-L1 blockade has shown to be an effective treatment approach in patients with advanced colorectal and non-colorectal dMMR/MSI-H cancers.^4–6^ Nevertheless, only a third of patients treated with anti-PD-1 monotherapy obtains a durable response. The anti-PD-1 antibodies pembrolizumab and dostarlimab have received regulatory approval for the treatment of patients with advanced non-colorectal dMMR/MSI malignancies.

A single arm non-randomized multi-cohort clinical trial in patients with advanced dMMR/MSI-H colorectal cancer has similarly suggested superiority of combined anti-PD-1/CTLA-4 blockade using nivolumab and ipilimumab compared to nivolumab monotherapy.^7^ Additionally, there is limited evidence that combined anti-PD-1/CTLA-4 blockade can overcome resistance in patients with MSI-H malignancies who progress on anti-PD-1 monotherapy.^8,9^

MoST-CIRCUIT is the first clinical trial to prospectively investigate immunotherapy using anti-PD-1/CTLA-4 blockade in patients with advanced dMMR/MSI-H non-colorectal cancers.

## Methods

### Study Design, Treatment and Participants

MoST-CIRCUIT was a multicentre open label phase 2 basket trial conducted at 17 Australian and New Zealand sites that enrolled 240 patients with selected advanced rare cancers. Eligible patients were aged 18 years or older and had a protocol defined advanced rare cancer including a dMMR/MSI-H non-colorectal cancer (cohort D). Patients had at least one measurable lesion according to Response Evaluation Criteria In Solid Tumour (RECIST) version 1.1 and an Eastern Cooperative Oncology Group (ECOG) performance status of 0 or 1. Other inclusion criteria were a life expectancy of three months or more as estimated by the treating physician (considering urgency for treatment, performance status, comorbidities) and adequate organ function. Patients could either be treatment naive or had received one prior systemic therapy for metastatic disease with a minimum washout period of 28 days before initiation of study treatment. Key exclusion criteria were active brain metastases and a history of autoimmune conditions. Archival tumour tissue, or a fresh tumour biopsy during screening, was required for explorative biomarker analysis.

The clinical trial protocol was reviewed and approved by the Institutional Review Board at Austin Health (Melbourne, Australia) and was undertaken in accordance with the Declaration of Helsinki and the guidelines of Good Clinical Practice. Written informed consent was obtained from all participants prior to enrolment into the study. Nivolumab and ipilimumab were administered intravenously at a dose of 3mg/kg over a period of 60 minutes and 1mg/kg over a period of 90 minutes, respectively, every three weeks for four doses (induction phase), followed by nivolumab monotherapy at a dose of 480mg every four weeks (maintenance phase) until disease progression or a maximum of two years after enrolment.

Tumour assessments were performed by radiological assessment (computer tomography of brain, chest, abdomen, pelvis) at baseline and then every 12 weeks during treatment or follow up. Tumour response was assessed according to Response Evaluation Criteria in Solid Tumours (RECIST) version 1.1. Clinical benefit rate has been defined as the percentage of patients that obtain an objective response or had stable disease for more than 12 weeks.

Safety analyses were performed on all patients who received at least one dose of study treatment. Laboratory monitoring and safety assessments were performed at baseline and every three to four weeks prior to treatment according to the study protocol. Adverse events were graded in accordance with the NCI Common Terminology Criteria for Adverse Events version 5.0 and collected during treatment and for 100 days after the last dose received.

### Outcomes

The co-primary endpoints were the proportion of patients with an objective response (complete or partial response) according to RECIST criteria and the proportion of patients alive and free of disease progression at six months (6-month-PFS). The secondary endpoints were median OS and PFS and treatment related toxicity.

### Testing for dMMR and MSI testing

Archival formalin-fixed paraffin-embedded tumour tissue was tested by each site’s the local NATA accredited pathology provider for the expression of mismatch repair proteins (MLH1/PMS2/MSH2/MSH6) by immunohistochemistry (IHC). Eligibility required the loss of at least one mismatch repair protein in the tumour tissue and/or microsatellite instability determined by PCR or NGS testing.^10^

### Tumour profiling

Tumour genomic profiling was performed using FFPE material by Omico’s Molecular Screening and Therapeutics Clinical trials & immunotherapy (MoST) framework up until December 30^th^ 2023 and subsequently Omico’s Cancer Screening Program (CaSP) (n=47), utilising comprehensive NGS panels including Trusight Oncology 500 (TSO500 [Illumina, San Diego, CA]) (n=34) and FoundationOne CDx (Foundation Medicine, Inc., Cambridge, MA) (n=13).^11^ Profiling of single nucleotide and indel variants (short variants) was then performed by assessing for variants in the 289 genes that were common to all three panels. Only those short variants that were assessed as either likely pathogenic or pathogenic were included in further analysis.

### Statistical analysis

The primary objective of the MOST-CIRCUIT study was to confirm the clinical efficacy of ipilimumab and nivolumab observed in CA209-538 (30% ORR). A prospective sample size calculation indicated that a minimum of 58 patients should be enrolled in each rare cancer ‘basket’ on MoST-CIRCUIT, assuming a significance level of 5% (Zα/2=1.96) and power of 80% (Zβ=0.84) to ensure sufficient probability of detecting a true event (p1=0.3) compared with an approximate 10% response (p2=0.1) to standard care chemotherapy; immunotherapy was not approved for any of the included histotypes at study conception. Due to competitive recruitment between the tumour cohorts (A-D) in the last six months of the trial, only 52 of the originally planned 60 patients were enrolled into cohort D (dMMR/MSI cancers) at the time of study closure. Descriptive statistics (median, confidence intervals) were performed using GraphPad Prism v10.0.0 software. Survival proportions were estimated using the Kaplan-Meier method. Association between response and median tumour mutational burden (TMB) was assessed using the non-parametric Mann-Whitney U test. Association of select mutations (PTEN, CTNNB1, IFNGR1/JAK-1 and B2M) and clinical benefit to treatment was analysed using the Fisher’s exact test. Analysis of genomic aberrations was performed in the R coding environment (version 4.4.0) heatmaps displaying aberrations implicated in mismatch repair and immune evasion displayed using pheatmap package (version 1.0.12).

Exploratory analyses were performed to assess the association between aberrations and clinical outcomes (6mPFS and ORR) were performed using Fisher Exact Test and Benjamini-Hochberg multiple hypothesis correction. The corrected p-value threshold for statistical significance was set at 0.05.

## Results

### Patient characteristics and Disposition

From August 2021 to February 2024, 52 patients with advanced dMMR/MSI non-colorectal malignancies compromising 17 different tumour types were enrolled. Patient and disease characteristics are detailed in Table 1. All patients had stage IV disease and half of the patients were treatment naïve, whilst the remaining having received at least one line of therapy for metastatic disease; only one patient had received prior anti-PD-1 monotherapy. Endometrial cancer was the most frequent tumour type (50%) followed by carcinoma of unknown primary (8%) and duodenal cancer (6%) (Table 2).

The predominant dMMR pattern was MLH-1/PMS2 loss (34 patients, 65%) followed by MHS2/6 loss (10 patients, 19%). An isolated PMS2 or MSH6 loss was seen in three and two patients respectively. Tumours from two patients were mismatch repair proficient on IHC but microsatellite unstable based on genomic testing and associated with a high TMB. A cholangiocarcinoma patient with insufficient tumour tissue had a microsatellite unstable tumour based on testing of circulating tumour DNA which revealed a concomitant PMS2 mutation. Ten patients (19%) had dMMR tumours which were microsatellite stable on corresponding genomic testing. The tumours of 17 patients (32%) harboured a genomic mutation in one of the MMR genes, suggesting that the dMMR status of tumours of the remaining patients was due to epigenetic alterations as (e.g. MLH1 promoter methylation). Thirteen patients (25%) had a known germline mutation in keeping with a Lynch syndrome (Table 1). 36 (69%) patients completed the induction treatment with four doses of nivolumab and ipilimumab, five (9.6%) patients progressed clinically prior to the first radiological assessment. Five (9.6%) patients discontinued treatment during the induction period due to immune related adverse events (irAEs) and three subsequently switched to single agent nivolumab maintenance therapy. Out of 36 (69%) patients who completed induction treatment, 33 (63%) patients entered into the maintenance phase with monthly nivolumab infusions and three (5.7%) patients came off study for progressive disease at their first radiological assessment at week 12 (**eFigure 1 in Supplement 1**).

### Efficacy

**Figure 1:**
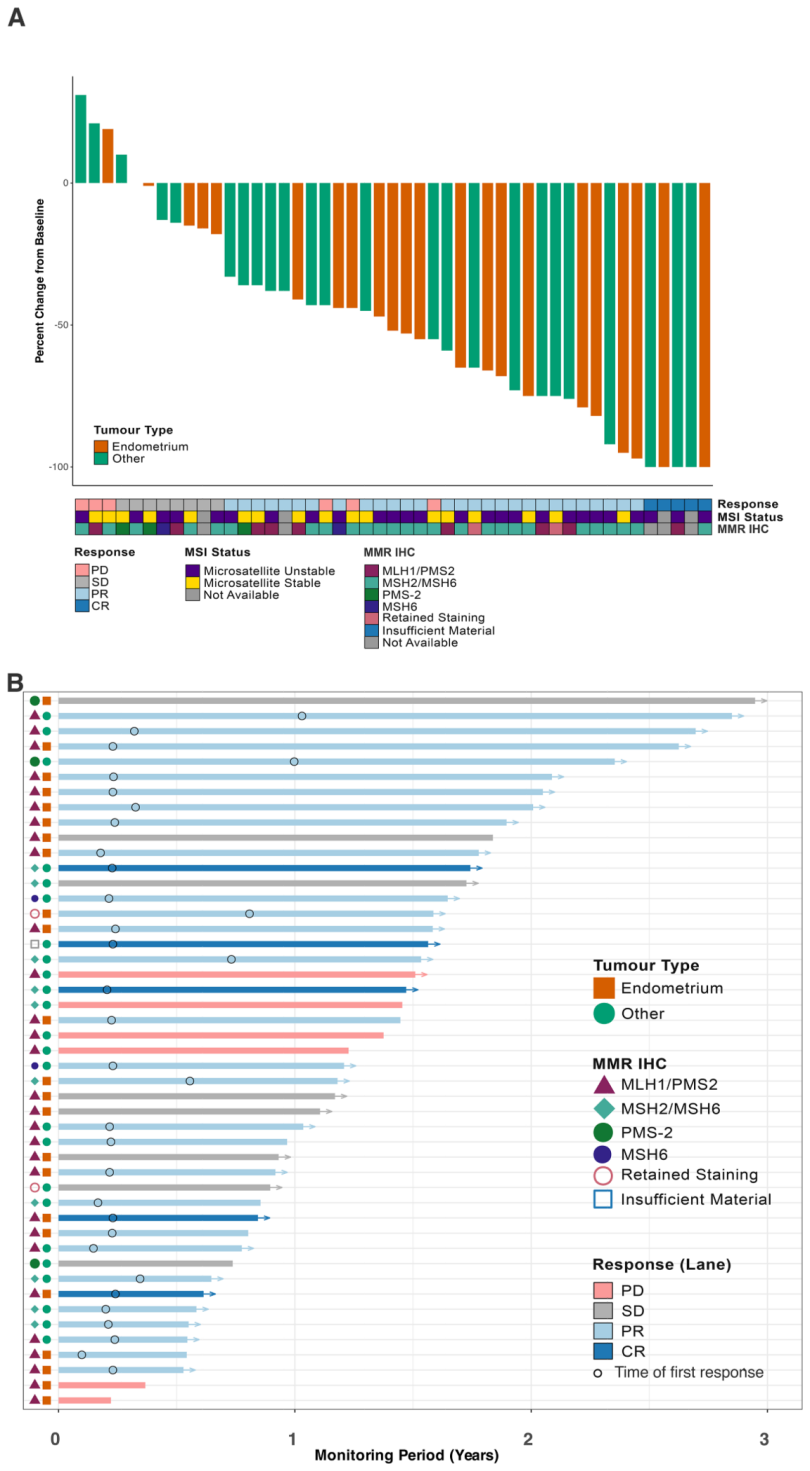
Efficacy outcomes. **A**, Waterfall plot of best percentage tumour change of target lesions from baseline depicting all patients who have undergone at least one radiological reassessment (n=47). Bar colours represent tumour type (endometrial or other), with other annotations as labelled. Arrows denote participants who remain alive and in follow-up. Acronyms: MSI = Microsatellite instability; MMR = DNA mismatch repair; IHC = immunohistochemistry. B, Swimmer plot depicting time to response and best overall response (lane colour) for the above patients (n=47 radiologically-evaluable patients)

Among the 52 patients enrolled into the trial in this cohort, five (10%) achieved a complete response and 28 (54%) a partial response leading to an objective response rate (ORR) of 63% (95% CI: 50 to 75%) (Figure 1A, **eTable 1, eTable 2 in Supplement 1**). The median duration of response has not been reached (range 2-31 months+) with 79% of responses being ongoing at the time of data analysis (Figure 1B). Eight patients (15%) experienced stable disease as their best response leading to a disease control rate of 79% (95% CI: 66 to 88%). Six patients (11%) had progressive disease at week 12 and another five patients (10%) progressed clinically prior to their first radiological assessment and came off study. Response did not differ between patients who were treatment naïve or pre-treated for metastatic disease: ORR 68% versus 63%. One patient was treated beyond disease progression that was detected on first restaging and continued on therapy close to a year until further progression.

Among the 26 patients with endometrial cancer, 15 (58%) obtained an objective response (95% CI: 39 to 74%) and five (22%) had stable disease leading to a disease control rate of 77% (95% CI: 58 to 89%) (Figure 1A, **eTable 1, eTable 2 in Supplement**). Two endometrial cancer patients progressed radiologically at week 12 and one clinically before their first restaging CT.

**Figure 2:**
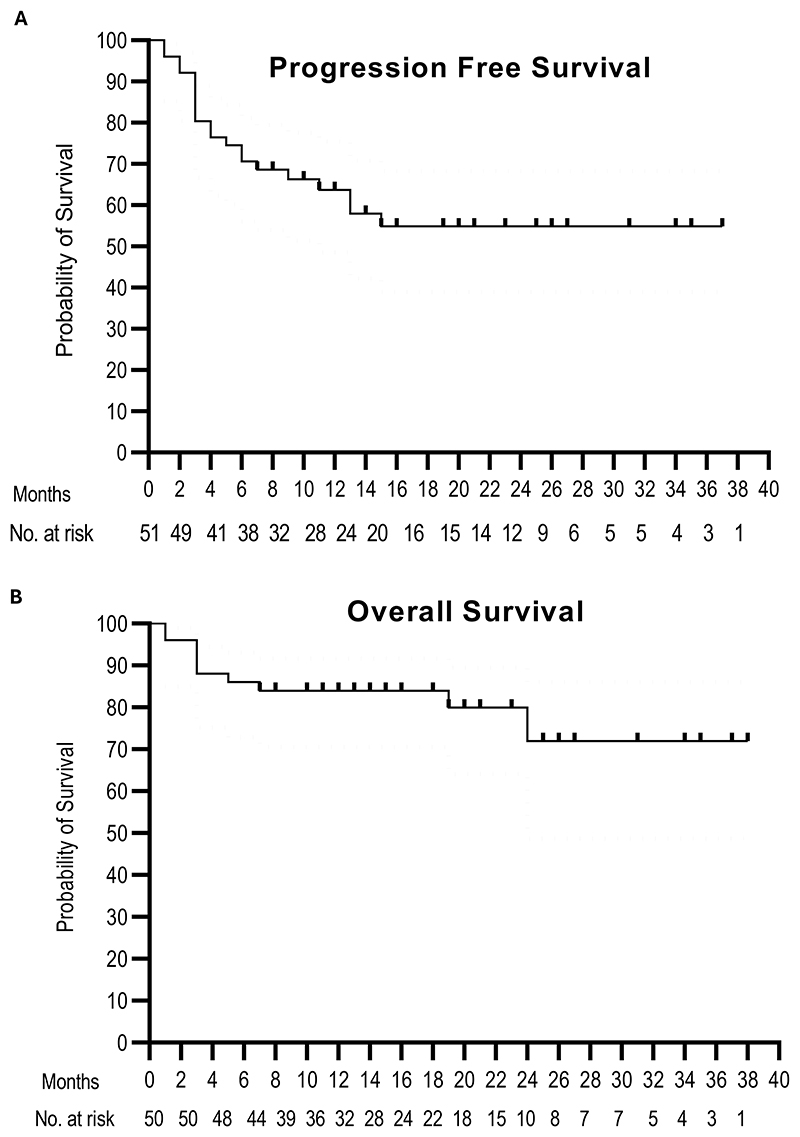
Survival outcomes. **A**, Kaplan-Meier graphical representation of progression-free-survival. **B**, Kaplan-Meier graphical representation of overall survival.

The six- months progression free survival was 71% for the overall cohort and 69% for patients with endometrial cancer respectively. The median progression-free and overall survival have not yet been reached with the median follow-up being 10.7 months (range 6.2 – 38 months) (Figure 2).

### Safety

39 (75%) of 52 patients experienced immune–related adverse events (irAE) of any grade (Table 3) with grade 3 or higher immune-related toxicity occurring in 12 (23%) patients with the most frequent irAE being hepatitis and entero-colitis. Mild infusion reactions and fatigue that did not relate to immune-mediated endocrinopathies were the only non-immune mediated adverse events being observed.

### Exploratory biomarker analyses

The objective response rate according to dMMR phenotype was 58% (95% CI: 42%, 74%) in MLH1/PMS2 deficient and 80% (95% CI: 31%, 83%) in MSH2/MSH6 deficient tumours. The response rate was 60% (95% CI: 31%, 83%) in the patients who had a dMMR tumour which was MSS on genomic testing. Due to the limited sample size, a formal comparison of these differences was not performed.

Genomic profiling was performed on tumours of 47 patients with the remaining five patients having insufficient tumour tissue for an analysis. An additional five tumour specimens were not suitable for TMB assessment due to low tumour purity. The median TMB (n=42) was 30.65 mutations per megabase (range 0.8-108.2 mut/Mb) and TMB was not associated with clinical benefit (**eFigure 2A/B in Supplement 1**). One of 4 patients with low TMB tumours (< 10/MB) achieved a response to treatment. The median TMB did not significantly differ according to dMMR subtype, 46.75 mut/MB for tumours with MSH2/6 loss and 30.05 mut/MB for MLH1/PMS-2 loss.

Genomic aberrations that are associated with resistance to anti-PD-1 monotherapy were detected in 29 (61%) of patients (**eFigure 2 in Supplement 1**).^12–15^ These included *JAK2* (21%), IFNGR1 (6%), B2M (6%), CTNNB1 (17%) and PTEN (51%) and none of these have been associated with resistance to combination therapy (**eFigure 2 in Supplement 1**). Additional mutations have been detected in 75 genes across the study cohort and none of these were associated with response. (**eFigure 3 in Supplement 1**).

## Discussion

MoST-CIRCUIT is the first trial to investigate immunotherapy using combined anti-PD-1/CTLA-4 checkpoint blockade in patients with advanced dMMR/MSI-H non-colorectal cancers. We observed a high rate of durable responses in our study population that compares favourably to published clinical trials using anti-PD-1 monotherapy.^5,6^ The objective response in the dMMR cohort of Keynote158 that investigated the anti-PD-1 antibody pembrolizumab was 30.8%, with 24% of patients being progression-free at 36 months. The most frequent enrolled tumour type as in this trial has been endometrial cancer, all patients had however received prior therapy for metastatic disease compared to only half of the patient population in our trial. Treatment response did not differ in our trial cohort between treatment naïve and pre-treated patients.

CheckMate142 was a non-randomized multi-cohort study that investigated monotherapy with nivolumab and combination treatment using nivolumab and ipilimumab in patients with advanced dMMR/MSI-H colorectal cancer. The objective response in the combination cohort was 65% compared to 31% in the nivolumab monotherapy group.^7^ The remarkably similar response rates that were observed in between our study population, the endometrial cancer subgroup and the combination therapy group of CheckMate142 suggests that dMMR/MSI-H may next to anti-PD-1 monotherapy serve as a tumour-agnostic biomarker for dual anti-PD-1/CTLA-4 treatment.^16^

Half of our trial population were patients with advanced endometrial cancer which is in keeping with the high frequency of microsatellite instability in this tumour type.^3^ Anti-PD-1/PD-L1 monotherapy demonstrated an objective response between 26 and 48% across several trials in this patient population and combination treatment of the anti-PD-1 antibody pembrolizumab with the multityrosine kinase inhibitor lenvatinib lead to an objective response of 41%.^17–20^ Recently, chemotherapy in combination with anti-PD-1/PD-L1 therapy has been established as a new standard first line therapy in patients with advanced endometrial cancer independent of their microsatellite status ^21,22^. Further clinical trials will be required to assess if the addition of chemotherapy to checkpoint blockade provides any additional benefit in the dMMR/MSI-H subgroup compared to checkpoint inhibitor therapy alone, the preferred treatment approach in patients with advanced dMMR/MSI-H colorectal cancers. In addition, an ongoing phase II randomized trial investigating nivolumab monotherapy compared to combination treatment with ipilimumab in advanced endometrial cancer will provide additional evidence for any potential difference in efficacy between combined checkpoint blockade and anti-PD-1/PD-L1 monotherapy (NCT05112601).

It has been reported that dMMR/MSI-H non-colorectal cancers demonstrate frequently atypical dMMR patterns including retained protein expression with concomitant microsatellite instability defined by genomic testing or classic mismatch repair protein loss with a concomitant microsatellite stable phenotype.^23^ We observed in 19% of patients of our study cohort a discordance between dMMR IHC testing and genomic analysis for microsatellite instability, an observation that is more frequently seen in dMMR non-colorectal cancers. The response rate in these patients did not differ from patients their tumours had concurrent test results.

A trial with the anti-PD-1 antibody pembrolizumab in dMMR endometrial cancer demonstrated a lower response rate in patients with tumours that were dMMR due to epigenetic changes compared to patients their tumours harboured a somatic or germline mutation in MMR encoding genes, this observation was linked to distinct immunological mechanisms that mediate anti-tumour immune responses in both subgroups.^24^ Additionally, clinicopathological features and the immune microenvironment has been shown to differ between both subgroups with tumours harbouring MMR gene mutations demonstrating an increased number of CD8 T cells compared to microsatellite unstable tumours caused by MLH-1 promotor methylation.^25,26^ We were unable to detect any differential response between both subgroups, which may be due to the limited number of patients that were enrolled into both trials or the use of combined anti-PD-1/CTLA-4 checkpoint blockade in our study compared to anti-PD-1 monotherapy in the former trial.

An association between a higher TMB and a treatment response to anti-PD-1 monotherapy has been demonstrated in dMMR/MSI-H colorectal, gastric cancer and other gastrointestinal malignancies.^14,27,28^ We did not observe any difference in the median TMB between responders and non-responders, which once again may relate to treatment with combined anti-PD-1/CTLA-4 checkpoint inhibition compared to anti-PD-1 monotherapy in the former trials. In addition, recent pre-clinical work demonstrated that clonal, but not subclonal TMB associates with treatment response in dMMR/MSI-H cancers potentially accounting for the observed differences.^29^

Genomic aberrations previously linked to resistance in dMMR/MSI-H tumours treated with anti-PD-1/PD-L1 monotherapy were detected in more than half of the study population^12–15,30^ but their presence did not influence response to therapy suggesting that combined anti-PD-1/CTLA-4 blockade may overcome these resistance mechanisms. This is in keeping with clinical observations that patients with dMMR malignancies progressing on anti-PD-1 monotherapy can obtain a subsequent treatment response to combined anti-PD-1/CTLA-4 checkpoint blockade.^8,9^ Mutations affecting genes of the IFN gamma pathway have been linked to primary and secondary resistance to checkpoint inhibition ^12,15^; in contrary, a recent large pan-cancer analysis of patients treated with checkpoint inhibitors demonstrated that gene mutations of the INF gamma pathway were positively associated with response.^31^ Further investigations will therefore be required to determine how these mutations shape the anti-tumour immune response and affect immune effector cells other than CD8 and CD4 T cells that can contribute to tumour eradication.^32^

A quarter of trial participants experienced high grade immune-related toxicity with the most common being entero-colitis and hepatitis. The frequency of severe irAEs was comparable to clinical trials using the same ipilimumab and nivolumab dosing and scheduling regimen in other malignancies.^33^

Limitations of the presented results are the modest size of the trial cohort and the single arm trial design that precludes a direct comparison to anti-PD-1 monotherapy. Recently, CheckMate 8HW, a randomized phase III trial that compared nivolumab and ipilimumab combination therapy to nivolumab monotherapy and chemotherapy in patients with advanced dMMR/MSI-H colorectal cancers demonstrated superiority of the combined treatment approach to anti-PD-1 monotherapy.^34^. It is unlikely that a similar trial will be conducted in dMMR non-colorectal cancers, our data however support the use of nivolumab and ipilimumab combination therapy as a treatment option in this patient population.

## Figures and Tables

**Table 1:** Demographics

Characteristic	N=52
*Age, years, median (range)*	62 (29-84)
*Gender*	
Male	11 (21%)
Female	41 (79%)
*ECOG performance status*	
0	18 (35%)
1	34 (65%)
*No. of prior systemic regimens*	
0	25 (48%)
1	19 (37%)
2+	8 (15%)
*dMMR subtype*	
MLH1/PMS2 loss	34 (65%)
MSH2/MSH6 loss	10 (19%)
PMS2 isolated loss	3 (6%)
MSH6 isolated loss	2 (4%)
Retained staining (MSI-H)	2 (4%)
No tissue, MSI on ctDNA	1 (2%)
*Lynch syndrome*	
Yes	12 (23%)
No	32 (62%)
Unknown	8 (15%)

**Table 2:** Tumour types

Tumour type	N=52
Endometrial cancer	
Endometrioid adenocarcinoma 17	
Poorly differentiated carcinoma 4	
Carcinosarcoma 3	26
Serous carcinoma 1	
Clear cell carcinoma 1	
Carcinoma of unknown primary	4
Duodenal cancer	3
Cholangiocarcinoma	2
Pancreatic cancer	2
Lung cancer	2
Gastric cancer	2
Ovarian cancer	2
Urothelial carcinoma	2
Breast cancer	1
Prostate cancer	1
Esophageal cancer	1
Leiomyosarcoma	1
Choriocarcinoma	1
Thyroid cancer	1
Cutaneous Squamous cell carcinoma	1

**Table 3:** Immune -Related Toxicity


Dermatological (Rash, Pruritus)	19 (36%)	2 (4%)
Endocrine		
Thyroiditis/Hypothyroidism	5 (10%)	1 (2%)
Hypophysitis	1 (2%)	0 (0%)
Adrenal Insufficiency	1(2%)	1 (2%)
Diabetes	0 (0%)	1 (2%)
Hepatitis	7 (13%)	3 (6%)
Enterocolitis/Diarrhea	13 (25%)	3 (6%)
Pancreatitis/Lipase increased	2 (4%)	1 (2%)
Arthritis/Arthralgia	9 (17%)	0 (0%)
Pneumonitis	1 (2%)	1 (2%)
Gastritis	0 (0%)	1 (2%)
Nephritis	1 (2%)	0 (0%)

## Key Points

### Question

What is the efficacy of nivolumab and ipilimumab combination treatment in patients with advanced dMMR/MSI-H non-colorectal cancers?

### Findings

52 patients with dMMR/MSI-H non-colorectal cancers were enrolled into this phase 2 nonrandomized clinical trial. An objective response rate of 63% and a six-month-progression-free survival of 71% was observed.

### Meaning

Nivolumab and ipilimumab has significant activity in dMMR/MSI-H non-colorectal cancers and represents next to anti-PD-1 monotherapy a treatment option.
